# Two-Dimensional Chiral Metasurfaces Obtained by Geometrically Simple Meta-atom Rotations

**DOI:** 10.1021/acs.nanolett.3c02168

**Published:** 2023-09-19

**Authors:** Dmytro Gryb, Fedja J. Wendisch, Andreas Aigner, Thorsten Gölz, Andreas Tittl, Leonardo de S. Menezes, Stefan A. Maier

**Affiliations:** † Chair in Hybrid Nanosystems, Nano Institute Munich, Department of Physics, Ludwig-Maximilians-Universität München, 80539 Munich, Germany; ‡ Departamento de Física, Universidade Federal de Pernambuco, 50670-901 Recife, PE, Brazil; § School of Physics and Astronomy, Monash University, Clayton, Victoria 3800, Australia; ∥ Department of Physics, Imperial College London, London SW7 2AZ, United Kingdom

**Keywords:** chirality, chiroptical response, dielectric metasurface, meta atom rotation, chiral arrangement

## Abstract

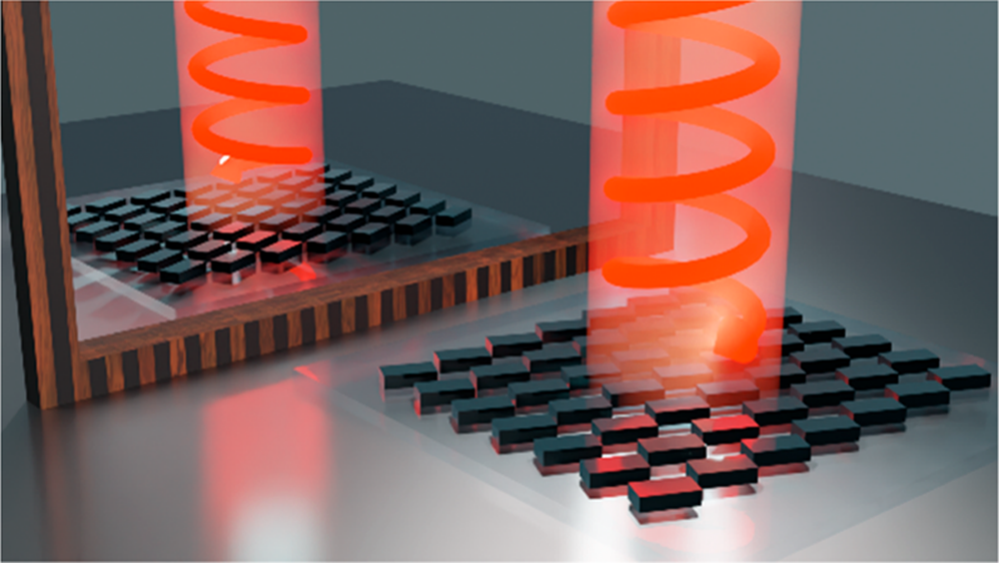

Two-dimensional chiral metasurfaces seem to contradict Lord Kelvin’s geometric definition of chirality since they can be made to coincide by performing rotational operations. Nevertheless, most planar chiral metasurface designs often use complex meta-atom shapes to create flat versions of three-dimensional helices, although the visual appearance does not improve their chiroptical response but complicates their optimization and fabrication due to the resulting large parameter space. Here we present one of the geometrically simplest two-dimensional chiral metasurface platforms consisting of achiral dielectric rods arranged in a square lattice. Chirality is created by rotating the individual meta-atoms, making their arrangement chiral and leading to chiroptical responses that are stronger or comparable to more complex designs. We show that resonances depending on the arrangement are robust against geometric variations and behave similarly in experiments and simulations. Finally, we explain the origin of chirality and behavior of our platform by simple considerations of the geometric asymmetry and gap size.

Chirality can be found on all scales from the chirality of spiral galaxies down to the fundamental biological building blocks of nature, such as amino acids, sugars, and DNA, where chirality can give rise to dramatic differences in protein function, cell communication, and organism health.^1,2^ In recent years, the field of chirality also attracted interest from researchers working in metasurfaces and nanophotonics, due to the possibility to manipulate the interaction with circularly polarized light, which can be exploited for optical components,^3,4^ to generate superchiral light fields,^5^ for materials with negative refractive index,^6^ or to enhance biosensing of chiral molecules.^7−9^ Chiral nanostructures have been fabricated at the single particle level^10−14^ or in metasurfaces using two-^7,15−19^ and three-dimensional meta-atoms.^3,20−22^ In particular, planar 2D chiral metasurfaces seem to contradict Lord Kelvin’s 1904 definition of chirality,^23^ since their mirror images can be made to coincide by rotational operations. In general, this definition applies very well to any kind of 3D object, such as a 3D helix or many chiral particles.^10,11,13,14^ It has been proposed that the origin of planar chirality results from differences at their interfaces, i.e., the presence of a substrate or fabrication defects at the surface that give the nanostructure a three-dimensional character.^24−26^ However, chiral 2D and 3D nanostructures behave differently in the chiroptical measurements. Typically, the chiroptical response of 3D structures is independent of the illumination side and is characterized by copolarized transmission,^26,27^ unlike 2D metasurfaces, for which the sign of the chiroptical response reverses when the structure is illuminated from the opposite side,^28^ and their chiroptical response is characterized by polarization conversion, i.e. cross-polarized transmission.^29^ The behavior of 2D chiral metasurfaces can thus be clearly differentiated from 3D objects and appears to contradict Lord Kelvin’s definition of chirality. To account for these differences, Schäferling et al. and others distinguish between *geometrical chirality* for objects that obey Lord Kelvin’s definition and *chirality* for objects that exhibit a near-field chiroptical response.^30,31^ Schäferling et al. also extend Kelvin’s definition to describe the chirality of 2D planar metasurfaces, which cannot be brought into coincidence with their mirror images if it is forbidden to lift the sample out of the plane. Moreover, they intuitively illustrate the strength of the chiroptical response by overlapping the unit cell with its mirror image and maximizing the asymmetry, i.e., minimizing the geometric overlap of both enantiomers. While this represents a very simple approach to design and optimize chiral 2D metasurfaces, currently used meta-atom designs are very complex and rather attempt to satisfy Kelvin’s geometric definition of chirality. Extensive efforts have been made to experimentally realize 3D helical shapes,^3,20^ but their fabrication at the nanoscale is only possible with sophisticated fabrication techniques.^21,22,32^ The same is true for other 3D approaches based on multilayer 2D systems,^33−38^ in-plane symmetry breaking,^27,39,40^ assembling achiral particles in a 3D architecture,^41−43^ or fabricating chiral single particles.^11−14^ For planar 2D metasurfaces, most meta-atom designs attempt to create a flat version of the helix, e.g. gammadions,^7,18,25,26,44^ Z-structures,^16,17^ spirals,^19,45^ or propeller-shaped nanostructures.^46−48^ However, a strong visual appearance of chirality is not necessarily correlated with enhanced chiroptical response, and the complexity of these designs is accompanied by a large number of geometric parameters, all of which affect the structures optical properties. This complicates their optimization, which is usually performed by numerical simulations or even using artificial intelligence.^49,50^ While such comprehensive approaches are important to maximize the chiroptical response to unprecedented levels, their complexity hinders their use in practical applications. For example, the desirable opportunity for the pharmaceutical industry to improve selective enantiomeric fabrication yield and biosensing of chiral molecules may require rather simple platforms that are easy to understand, modify, fabricate, and use by researchers from a variety of fields. Moreover, the experimental response of metasurfaces always represents an ensemble measurement across all illuminated meta-atoms and may differ from simulations, because fabrication will always lead to geometrical variations between individual meta-atoms.^51^ The effect of such geometric variations of complex structures with a large parameter space is, at least in our experience, hard to understand and often leads to completely different resonances in experiment and simulation; hence, the simulated resonances are difficult to realize experimentally.

Here, we experimentally and numerically present one of the geometrically simplest 2D planar chiral metasurface designs possible. The metasurface consists of rods as meta-atoms that are intrinsically achiral. Chirality is created by rotating the meta-atoms through an angle of rotation, making their arrangement in a metasurface chiral.^52−54^ Although the proposed design is geometrically simple, the measured and simulated far-field response is stronger, or at least comparable to those shown by many more complex designs.^27,40,44^ While rotated dielectric bars are very prominent in the field of beam shaping and focusing by utilizing the Pancharatnam-Berry phase,^55^ their chiral response is usually overlooked. To date only theoretical work exists on rotation induced chirality by utilizing achiral plasmonic gold dimers and rods^52,53^ or by using achiral plasmonic hole arrays in gold films.^54^ We generalize this concept for dielectric metasurfaces, which may be more suitable for chiral applications, such as biosensing, since plasmonic nanostructures can suffer from intrinsic losses.^8^ We follow the rotation angle dependent evolution of our resonances with circularly and linearly polarized illumination and investigate the effect of the geometric asymmetry, the gap sizes between our structures, and the chiral near-fields on the chiroptical response. Moreover, our analysis shows that a particular resonance, which depends mainly on the arrangement, i.e. pitch and gap size, is barely affected by structural deviations and occurs with the same modulation in experiment and simulation, contrary to resonances depending on the dimensions of the meta-atoms. This shows that numerical optimization of complex designs may often not find correspondence in experiments and that it is necessary to incorporate experimentally realistic variations in geometry into the simulations.

Our simple metasurface platform consists of rectangular rods with a length of *l* = 473 nm, a width of *w* = 247 nm, and a height of *h* = 127 nm made from amorphous silicon (a-Si) arranged in a square lattice with a pitch *p* = 540 nm. An overview of the platform is shown in Figure 1. The metasurfaces are fabricated on a glass substrate covered with a 18 nm thick layer of transparent indium tin oxide (ITO), which allows the substrate to be conductive to facilitate scanning electron microscopy (SEM), but has no other function and is not influencing the chiroptical response of our platform. For the achiral case, the rods are symmetrically arranged along the *x*- and *y*-axes (coordinate system in Figure 1a), and they have mirror symmetry in the *xz*- and *yz*-planes through the origin of each meta-atom. The meta-atoms also have C2 rotational symmetry, which is not further relevant since this also applies to the chiral metasurfaces. To create chirality, each rod is rotated around its center with a rotation angle θ. As each meta-atom is rotated, the entire arrangement loses its mirror symmetry in *xz*- and *yz*-planes, making the metasurface chiral as long as the rotation angle for the square lattice is not a multiple of π/4 (45°), where the *xz*- and *yz*-mirror symmetry is restored when the coordinate system is rotated with the same angle. An intuitive graphical illustration of this behavior can be found in Figure S1 and in ref (54), where the case of a hexagonal lattice is also discussed.

**Figure 1:**
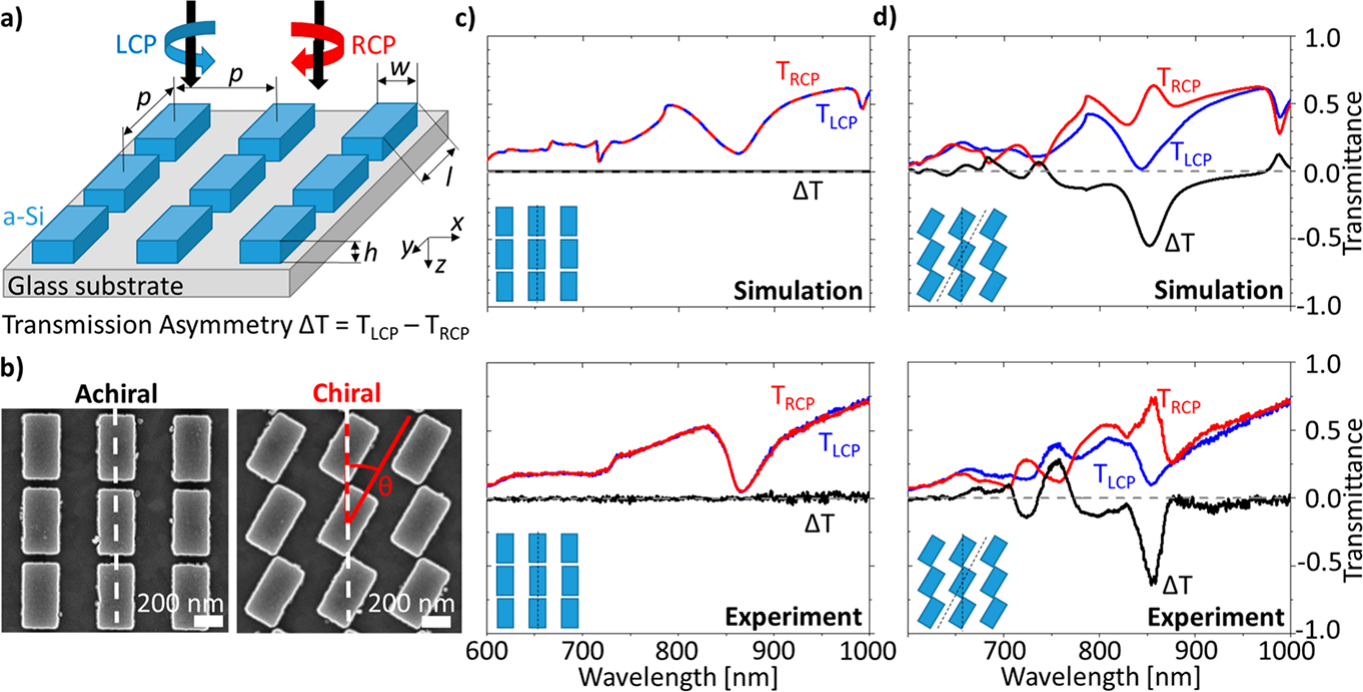
Concept of generating 2D chiral metasurfaces by rotation of achiral meta-atoms. (a) Schematic representation of the relevant geometrical parameters. The metasurfaces of rods of amorphous Si (a-Si) with length (*l*), width (*w*), and height (*h*) are arranged in a square lattice with pitch (*p*) on a glass substrate covered with 18 nm of indium tin oxide (ITO). Optical characterization is performed by illuminating the metasurfaces with left and right circular polarized light, LCP and RCP, respectively, and by measuring their transmission. (b) Scanning electron microscopy (SEM) images of an achiral (left) and a chiral (right) metasurface with a rotation angle of 25°. The chirality results from the rotation of the achiral meta-atoms by a rotation angle θ. (c, d) Simulated (top) and experimental (bottom) transmission and transmission asymmetry (Δ*T* = *T*_LCP_ – *T*_RCP_) spectra of the achiral (c) and chiral (d) metasurfaces with a rotation angle of 25°. The chiral (d) metasurface shows clear chiral resonances in the visible and near-IR region.

We investigate experimentally and numerically a series of metasurfaces with rotation angles varying from −45° to +45° in 5° steps. The initial design of the planar 2D metasurface was performed numerically to ensure that the resonances occur in the optical regime (details in SI, Section 1). Next, we fabricated the metasurfaces (details in the SI, Section 2) from a-Si films deposited by plasma-enhanced chemical vapor deposition (PE-CVD), patterned with electron beam lithography (EBL) and etched with a chromium hard mask by reactive ion etching (RIE). Subsequently, the metasurfaces were characterized using SEM and atomic force microscopy (AFM) and the numerical simulations were repeated by using the experimentally measured dimensions. Figure 1b shows SEM images of the achiral (left) and chiral (right) metasurfaces with a rotation angle of 25°. SEM images of all other metasurfaces at different magnifications can be found in Figure S2, and the AFM data in Figure S3.

The chiroptical response was measured experimentally with a custom-built microscopy setup by illuminating the metasurfaces with left and right circularly polarized light, LCP and RCP, respectively (details in Supporting Information (SI), section 3). The transmission asymmetry Δ*T* = *T*_LCP_ – *T*_RCP_ was calculated from the difference in the LCP and RCP light transmission. Each measurement was performed twice with the substrate rotated by 90° (details in SI, Section 4 and Figure S4). Figure 1c,d shows the simulated (top) and experimental (bottom) transmission spectra of the achiral and chiral metasurfaces with a rotation angle of 0° and 25°, respectively. In general, the spectra show excellent agreement with some minor discrepancies between experiment and simulation. Starting with the achiral metasurface (Figure 1c), a strong resonance is clearly visible at 850 nm in experiment and simulation, with the simulated one being broader than the experimental. At 980 nm, another resonance is visible in the simulation not observed in the experiment and which we do not consider further in the manuscript. A reason may be that this wavelength is outside the range of the quarter-wave plate or that the resonance is shifted to wavelengths > 1000 nm, which is outside the range of our spectrometer. For the achiral case, the transmission for LCP and RCP is identical, resulting in zero transmission asymmetry. For the chiral metasurface, a clear chiroptical response is seen in both experiment and simulation. At 850 nm, the dip in *T*_LCP_ remains similar to the achiral case, while *T*_RCP_ reverses its behavior and shows a peak with increased transmission, leading to a strong chiral resonance. Below 800 nm, the interpretation is more difficult because there is a large number of spectral features, and, in general, all CD features are less pronounced in the simulation. Apart from their modulation strength, all peaks and dips occur at the same wavelengths, except for the experimental peak in *T*_LCP_ at 750 nm, which does not appear in the simulation.

Next, we varied the rotation angles from −45° to +45° in 5° steps and tracked the evolution of each resonance leading to the appearance of chirality. The evolution of Δ*T* for negative and positive rotation angles is shown in Figure 2a and b, respectively, while the transmission spectra are shown in Figures S5 and S6. Since positive (clockwise) and negative (counter clockwise) rotations are mirror images, their Δ*T* spectra are perfectly mirrored and only *T*_LCP_ and *T*_RCP_ are exchanged. At each rotation, the chiral resonances appear at the same wavelengths with no obvious shifts, but the modulation becomes stronger until it reaches a maximum at ±25°, from where it decreases again and is achiral at ±45°.

**Figure 2:**
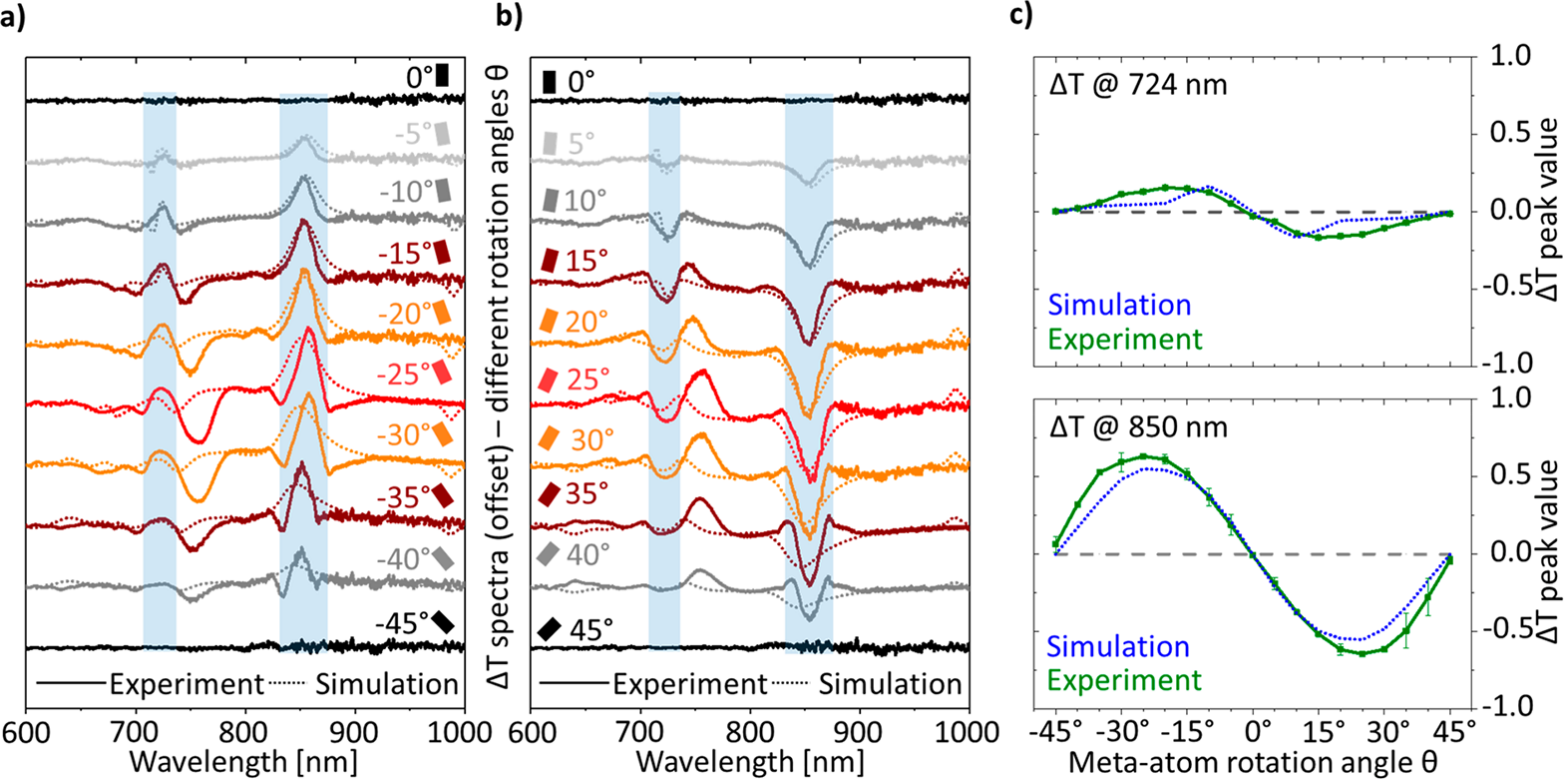
Experimental and simulated spectra of transmission asymmetry Δ*T* of 2D metasurfaces with different rotation angles. (a,b) Experimental and simulated spectra of 19 metasurfaces with rotation angles varying in 5° steps from 0° to −45° in (a) and from 0° to 45° in (b). The rotation angles are indicated by small schematic structures corresponding to the color scheme of the spectra. The structure is achiral for 0°, 45°, and −45° (Δ*T* = 0). For the chiral metasurfaces, the Δ*T* spectra are perfectly mirrored for ± rotations. (c) Comparison between experiment (green line) and simulation (blue line) of the maximum peak values at the first peak at 724 nm (top) and the second peak at 850 nm (bottom).

To explain the behavior of the observed resonances in more detail, we characterized all metasurfaces with horizontal and vertical linear polarizations, HP and VP, respectively (SI, Section 5). We simulated the optical response of the achiral sample with small parameter sweeps (Figure S7), and we followed the evolution of wavelength shifts during the rotation of the meta-atoms (Figure S8). The results show that resonances occurring below 800 nm are strongly correlated with the dimensions of the structures, i.e., length and width, while resonances above 800 nm are more related to the arrangement in the metasurface, i.e., pitch and gap size. Here we will focus on the two main resonances at 850 and 724 nm, which are clearly present in both simulation and experiment. We neglect the resonance at 750 nm since its experimental modulation strength mainly originates from a peak in *T*_LCP_ that we do not observe in the simulation (see Figure 1d). Figure 2c shows the maximum values for these two resonances for each rotation angle. It can be clearly seen that the resonance at 850 nm shows excellent agreement between simulation and experiment (Figure 2c bottom) with the experimental values slightly outperforming the simulation ones and the peak values showing a symmetric shape, reaching the highest Δ*T* values at rotation angles of ±25°. The better performance of the experimental values results from small spectral shifts in the experiment. In the experiment, the transmission dip and peak for T_LCP_ and T_RCP_, respectively (Figures 1d and S6), occur at the exact same wavelength, while in the simulation they are slightly shifted by around 10 nm, leading to a weaker but broader modulation. At 724 nm, the experimental results (Figure 2c top) deviate from the simulated ones, and we observe weaker resonance strengths at ±25°, which we analyze in more detail in the next sections. The maximum values exhibit an asymmetric shape with the highest Δ*T* for rotation angles of ±10°.

Following, we simulated the normalized chiral near-field plots (C/C_0_) for the metasurface with a rotation angle of 25° (Figure 3). The chiral near-field plots illustrate at which locations of the structure the enhancements are the strongest and can be calculated from the simulated electric and magnetic near-field plots as follows
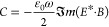
1where *C* is the optical chiral density, ε_0_ is the vacuum permittivity, ω is the angular frequency of light, and  is the scalar product of the complex conjugate of the electric field and the complex part of the magnetic field.^30^Equation 1 is a general requirement for resonances to exhibit a chiroptical response and leads to no chirality when the electric and magnetic field vectors are perpendicular to each other, i.e., their scalar product becomes zero, and leads to strong chiral densities when they have parallel components.^30,44^ For normalization, the optical chiral density (*C*) of the metasurface was divided by the chirality of the illumination source (*C*_0_). The corresponding electric and magnetic near-field plots are shown in Figure S9. It is clear that the chiral near fields are strongly confined to the structure at the resonance at 724 nm (Figure 3a). In comparison, the chiral near-fields at 850 nm are less pronounced inside the structure, while the enhancement outside the structure is similar (Figure 3b). Interestingly, even though the chiral fields at 850 nm seem to be much weaker, the simulated transmission difference still shows a larger magnitude (Figure 1c).

**Figure 3:**
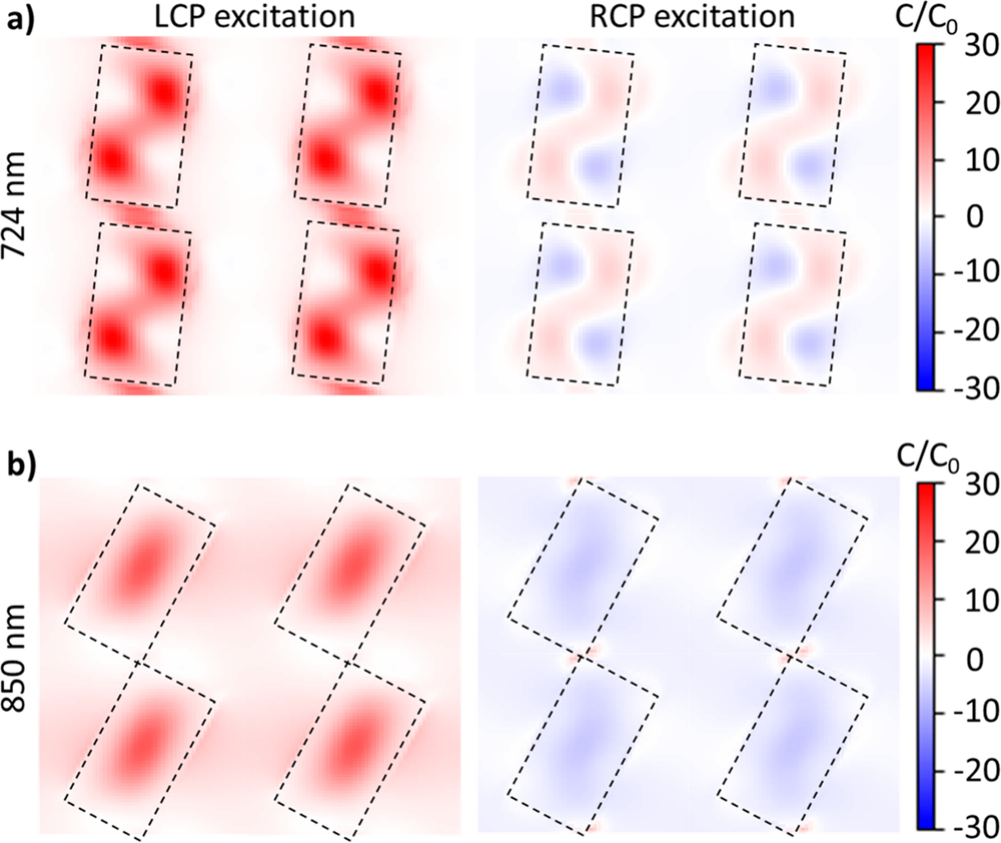
Chiral near-field plots for the metasurfaces with a rotation angles of 10° and 25° at their resonance wavelength. Chiral near-field plots at resonance wavelength for the metasurface with rotation angles of 10° (a) and 25° (b) for the LCP (left) and RCP (right). The near-fields at 724 nm are strongly confined inside the structure, in contrast to the chiral near-fields at 850 nm, which are less pronounced inside but of similar magnitude outside the structure.

We further characterized the metasurface with the highest transmission asymmetry, i.e., with a rotation angle of 25°, by analyzing the polarization of the transmitted light. Measuring the cross- and co-polarized transmissions allows to distinguish between the effects of 2D- and 3D-chirality, respectively.^27,29^ The results are shown in Figure S10 and clearly show dominating polarization conversion, especially for the resonance at 850 nm, i.e., stronger cross-polarized transmission induced by the in-plane mirror symmetry of our 2D metasurface. In contrast, the weaker copolarized transmission indicating 3D chirality, might be caused by the presence of the glass substrate, which formally breaks the out-of-plane symmetry.

Next, we incorporated slight variations in the geometric dimensions of the metasurface in our simulations (Figure 4). Figure 4a,b compares nine individual Δ*T* spectra for variations of ±5 nm in length and width. For the experimentally measured resonance wavelength at 724 nm, these small shifts in geometry already cause a difference of >0.2 in the transmission asymmetry, while the resonance at 850 nm varies by only ∼0.05. To reflect the experimentally realized dimensions, we varied the width and length in steps of 1 nm. Figure 4c,d shows the corresponding color contour plots of the Δ*T* values at the experimentally measured resonance wavelengths. It can be clearly seen that the resonance at 850 nm is stable over a wide range of geometric variations, presenting a transmission difference varying only by up to 0.1 for changes in length and width of ±10 nm, while the resonance at 724 nm is less stable, with Δ*T* varying by up to 0.3 and even changing the sign of the chiroptical response. This explains the differences between simulation and experiment (Figure 2), since the experiment is a weighted ensemble measurement over all of these different geometries. We point out that our fabrication with EBL yielded quite accurate results with realized gap sizes of ∼14 nm (Figure S11) and a standard deviation of the structures dimensions of only 1% in length and 2% in width (shown as white dashed rectangles in Figure 4c,d). This underlines the importance of accounting for geometric variations in the development of metasurfaces through numerical simulations. This is particularly relevant for chiral metasurfaces, since the chiroptical response is calculated from two individual spectra (LCP and RCP illumination) and even small shifts and variations in modulation can lead to strong differences in between experiment and simulation for their transmission asymmetry Δ*T* or circular dichroism (CD) spectra.

**Figure 4:**
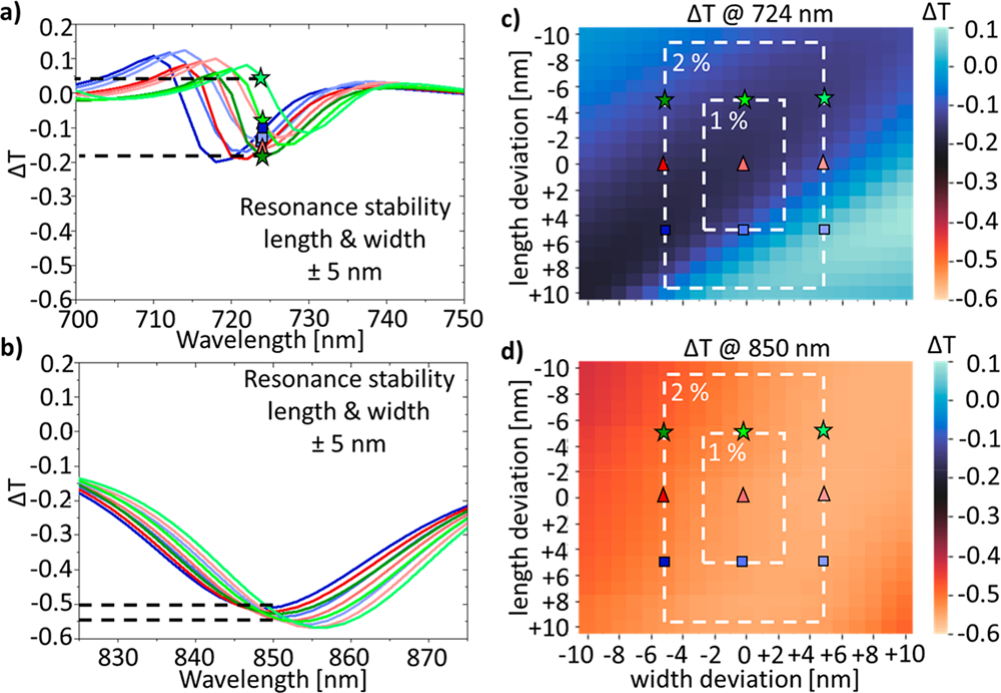
Resonance stability with geometric variations for the chiral metasurfaces. (a,b) Simulated Δ*T* spectra for slight variations in length and width by ±5 nm for both resonances for the metasurface with rotation angle of 10° (a) and 25° (b). The exact dimensions for each color are given in c,d. The dashed black lines illustrate the resulting variation in modulation strength that affects the experimental realization. (c,d) Color maps of the stability of the resonance with geometric variations in length and width in 1 nm steps for the metasurface with rotation angles of 10° (c) and 25° (d). The magnitude of the geometric variations corresponds to the standard deviation of the length and width measured in the experimental realization, i.e., about 1% in length and 2% in width (shown as white dashed rectangles). The markers indicate the position for the spectra shown in a) with a deviation of ±5 nm. For clarity, the markers are not shown in b).

Finally, we investigated why and for which rotation angles our simple chiral metasurface platform shows its maximum chiral response, i.e., 25° for the resonance at 850 nm. We found a good correlation between the amplitude of the transmission difference Δ*T* (Figure 2c) to the geometric asymmetry and the gap size. To quantify the geometric asymmetry, we calculated the geometric overlap^30^ between the mirror images of our meta-atoms with different rotation angles and analyzed at which rotation angles the geometric overlap of the structures is minimal, i.e., the asymmetry is maximum (graphic representation and more details in Figure S12). At ± 45° and 0°, the structures can be brought to perfect match, resulting in zero asymmetry and zero chiroptical response. For rods, the asymmetry shows symmetric behavior with a maximum value at 22.5°, which is quite close to our experimental results showing maximum chirality at 25°. In addition, the gap between our structures becomes minimal at 27.5°. To decouple both geometric asymmetry and gap size, we simulated rods with different widths, which changes the angle at which the smallest gap sizes occur. Indeed, the maximal chiroptical response shifts to smaller angles in correlation to the gap size as the width is decreased (Figure S13). We therefore conclude that the global properties of our platform are dominated by the geometric asymmetry, i.e., the platform cannot be chiral when there is a mirror symmetry (0° and ±45° for the square lattice), while the gap size mainly determines the strength of the chiral resonances. In addition to these two dominating factors, there can be strong local enhancements due to parallel components of the electric and magnetic field, which can occur quite randomly depending on the structure dimensions and rotation angle. We believe that the simulated maximum chiroptical response for the resonance at 724 nm for rotation angles of ±10° (Figure 2c, top and Figure 3a) may arise from such local enhancements.

In conclusion, we presented a geometrically simple chiral metasurface platform consisting of rectangular rods. Chirality is obtained by rotating each meta-atom, resulting in a 2D planar chiral metasurface as long as the rotation does not allow for out-of-plane mirror symmetries. Our approach completely avoids the utilization of complex meta-atom designs, which have a large parameter space and are therefore difficult to optimize and realize experimentally. Our analysis shows that chiral resonances depending on the arrangement, i.e., pitch and gap size) are stable. Finally, we show that the strength of the chiroptical response caused by the rotation of meta-atoms can be explained well by a convolution of maximizing geometric asymmetry and minimizing the gap size between the meta-atoms.

## Supporting Information Available

Materials, experiments, simulations: SEM images, AFM measurements, processing of optical data, transmission spectra for all rotation angles with different polarizations and with parameter variation, and schemes illustrating platforms behavior (PDF)
