# Supplementary material for: Two-Dimensional Chiral Metasurfaces Obtained by Geometrically Simple Meta-atom Rotations
Source: Nano Lett. 2023 Sep 19;23(19):8891–7. doi: 10.1021/acs.nanolett.3c02168 (PMC10571149; doi:10.1021/acs.nanolett.3c02168)
Supplement: Supplementary file 1 — nl3c02168_si_001.pdf [file nl3c02168_si_001.pdf]

Supporting Information

Two-Dimensional Chiral Metasurfaces obtained by Geometrically Simple Meta-atom Rotations

Dmytro Gryb[1,#], Fedja J. Wendisch[1,*,#], Andreas Aigner[1], Thorsten Gölz[1], Andreas Tittl[1], Leonardo de S. Menezes[1,2], Stefan A. Maier[3,4,1]

[1] Chair in Hybrid Nanosystems, Nano Institute Munich, Department of Physics, Ludwig-Maximilians-University Munich, 80539 Munich, Germany

[2] Departamento de Física, Universidade Federal de Pernambuco, 50670-901 Recife-PE, Brazil

[3] School of Physics and Astronomy, Monash University, Clayton Victoria 3800 Australia

[4] Department of Physics, Imperial College London, London SW7 2AZ, United Kingdom

*Fedja.Wendisch@lmu.de

# These authors contributed equally

TABLE OF CONTENT:

Materials & Solutions 3

Figure S1: Intuitive graphical representation explaining the chirality of the 2D chiral metasurface platform. 4

Section 1 - Details on the electromagnetic simulations 5

Section 2 - Details on the fabrication of the metasurfaces 7

Figure S2: Scanning Electron Microscopy (SEM) images of the fabricated metasurfaces. 8

Figure S3: Atomic Force Microscopy (AFM) images. 9

Section 3 - Details on the optical setup 10

Section 4 - Details on the optical characterization 12

Figure S4: Processing of the optical characterization. 12

Figure S5: Simulated transmission spectra for LCP and RCP illumination for all rotation angles. 13

Figure S6: Experimental transmission spectra for LCP and RCP illumination for all rotation angles. 14

Section 5 – Excitation with linear polarization. 15

Figure S7: Parameter variation of the structures dimensions with horizontal and vertical linear polarization. 16

Figure S8: Simulated transmission spectra for horizontal and vertical linear polarisation. 18

Figure S9: Electric and magnetic near-field plots for the metasurfaces with rotation angle of 10° and 25° at their resonance wavelength. 19

Figure S10: Cross- and co-polarized transmission and transmission asymmetry. 20

Figure S11: Gap sizes in the experimental realization. 21

Section 6 – Geometric asymmetry. 22

Figure S12: Asymmetry calculation of the metasurfaces with different rotation angles. 22

Figure S13: Comparison of the chiroptical response with the geometric assymetry and gap size for different meta-atom rotation angles and widths. 23

References 25

Materials & Solutions

All chemicals and solutions were used without further processing unless noted otherwise. Acetone (for HPLC, > 99.8 %), chromium etchant (standard), 2-propanol (IPA) (ACS reagent, > 99.8 %), were purchased from Sigma Aldrich. 495 polymethacrylate (PMMA) A4, 950 PMMA A4 were purchased from Kayaku, Advanced Materials. 950 PMMA A4 was mixed 1:1 with anisole to get 950 PMMA A2. E-Spacer 300Z was purchased from Showa Denko Europe GmbH. Anisole (for synthesis) was purchased from Merck. Microposit Remover 1165 was purchased from DUPONT. Methylisobutylketone (MIBK) was purchased by Technic. Glass substrates with 18 nm indium tin oxide (ITO) were purchased from Nanoscribe.

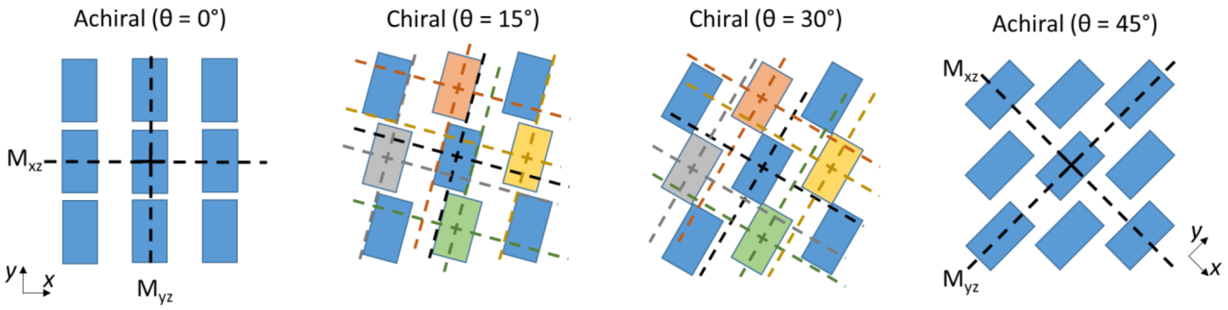

Figure S1: Intuitive graphical representation explaining the chirality of the 2D chiral metasurface platform.

The metasurface consists of rectangular rods that are inherently achiral and each meta-atom possesses C2 rotational symmetry as well as M_{xy}, M_{xz}, M_{yz} mirror plane symmetries. To create a chiral metasurface, each meta-atom is rotated around its center with a rotation angle θ. In the achiral cases (θ = 0°, 45°) the M_{xz} and M_{yz} mirror planes of each meta-atom coincide with the M_{xz}, M_{yz} of the other meta-atoms in the arrangement. In the chiral cases (θ ≠ 0°, 45°) the M_{xz}, M_{yz} mirror planes of each meta-atom do not coincide with the mirror planes of the other meta-atoms, so their arrangement is chiral. Chiral configurations are illustrated for two cases (θ = 15°, 30°), where the mirror planes of five individual meta-atoms are shown with different colors.

Section 1 - Details on the electromagnetic simulations

Simulations were performed using the commercially available finite-difference time-domain (FDTD) simulation package from Lumerical Inc. One unit cell was simulated using periodic boundary conditions in the *x*- and *y*- plane, while perfectly matched layers (PML) were used along the *z*-axis. Circularly polarized light was simulated with two sources of linearly polarized plane waves whose polarizations were perpendicular to each other and with a relative phase difference of 90° (RCP) or -90° (LCP). The plane waves were located 2.7 μm above the substrate and propagated in the *z*-direction. The dimensions of the meta-atoms were obtained from SEM and AFM measurements (Figure S2 and S3, respectively). The meta-atom was placed on a 75 μm thick glass (SiO_2) substrate with an 18 nm thick ITO layer. A refined mesh with dimensions 540 x 540 x 300 nm^3 and a mesh size of 10 nm was placed around the meta-atom. The transmission was modeled with a 2D power monitor located 5 μm below the glass substrate. The location of the transmission monitor and the relatively large thickness of the glass substrate (75 μm) were necessary to properly take into account diffracted waves in the simulations. For a metasurface with a pitch of 540 nm on glass, the diffraction cutoff is about 810 nm. This means that, below 810 nm, these diffracted waves are trapped in the glass substrate, hence the monitor must be placed outside of the glass substrate to reflect the experimental conditions.

For co- and cross-polarization components in the simulation, we used a Transmission Monitor. This monitor can output complex vector field components in the plane of observation. To obtain the polarization components, we applied a Jones matrix for circular polarization to a vector of electric field and then used square of this quantity summed for all points of the plain in order to get the intensity of the field. This value was normalized to initial light source intensities.

For all simulations, the spectral resolution was set to 2 nm. The dielectric constants of SiO_2 were used directly from the Lumerical materials library.[1] Moerland and Hoogenbooms data were used for ITO.[2] The refractive indexes of the Si rods were taken from in-house white-light ellipsometry data.

Section 2 - Details on the fabrication of the metasurfaces

Fabrication was performed similarly to other studies on chiral metasurfaces and has been reported before with minor changes in parameters.[3] Glass substrates coated with 18 nm of ITO were purchased commercially from Nanoscribe and cut into smaller pieces using a glass cutter. Prior to amorphous silicon (a-Si) deposition, the glass substrates were cleaned by sonication in acetone and isopropanol for 3 min each, dried with N_2, then treated with an oxygen plasma (Diener Femto, 100W, 20 mL/min; 3 min). Subsequently, 126 nm a-Si were deposited using plasma-enhanced chemical vapor deposition (PE-CVD) from Oxford Instruments (PlasmaPro 100). Deposition was performed for 8 min and 50 sec at 250 °C from silane (SiH_4) with a flow of 500 sccm, a chamber pressure of 1000 mTorr and power of 10 W.

Electron beam lithography (EBL) was performed using a spin-coated PMMA bilayer as electron-senstive resist. The first layer (PMMA 495k A4) was spun at 5000 rpm for 1 min (acceleration 1000 rpm/sec) and baked on a hot plate at 170 °C for 3 min. The second layer (PMMA 950k A2) was spun with 3000 rpm for 1 min (acceleration 1000 rpm/sec) and also baked in the same way. After PMMA, an e-spacer (electrification dissipating material) was spun at 2000 rpm for 1 min (acceleration: 1000 rpm/sec) to prevent charging during EBL. EBL was performed using an eLine Plus from Raith Nanofabrication with an acceleration voltage of 30 kV, an aperture of 20 μm, and a working distance of 10 mm. After EBL, the e-spacer was removed by rinsing the substrates in MilliQ-H_2O and developed in a 3:1 mixture of isopropanol and methylisobutylketone (MIBK) for 55 sec. Finally, the samples were rinsed with isopropanol and dried with N_2.

Next, 26 nm chromium were deposited using electron beam evaporation at a rate of 0.08 nm/sec at a pressure of ~10^{-6} mbar. PMMA was then removed (lift-off) by immersing the samples in Microposit Remover 1165 for 17 hours. Mild sonication (80 kHz, 30 % Power) was applied for

60 sec to remove all PMMA-Cr residues. Reactive ion etching (RIE) was performed using a PlasmaPro100 from Oxford Instruments. RIE was performed with a mixture of 7 sccm Ar and 20 sccm Cl_2 for 1 min and 12 sec with HF (high frequency) of 20 W and ICP (inductive coupled plasma) of 200 W at a pressure of 2 mTorr. Finally, the Cr was removed using a commercial Cr Etchant for 3 min.

SEM images of the fabricated metasurfaces are shown in **Figure S2**. At least 10 meta-atoms were measured with the free software ImageJ to determine the lateral dimensions, i.e. length *l*, width *w* and pitch *p*. The height of the metasurfaces was determined with atomic force microscopy (AFM) (see **Figure S3**).

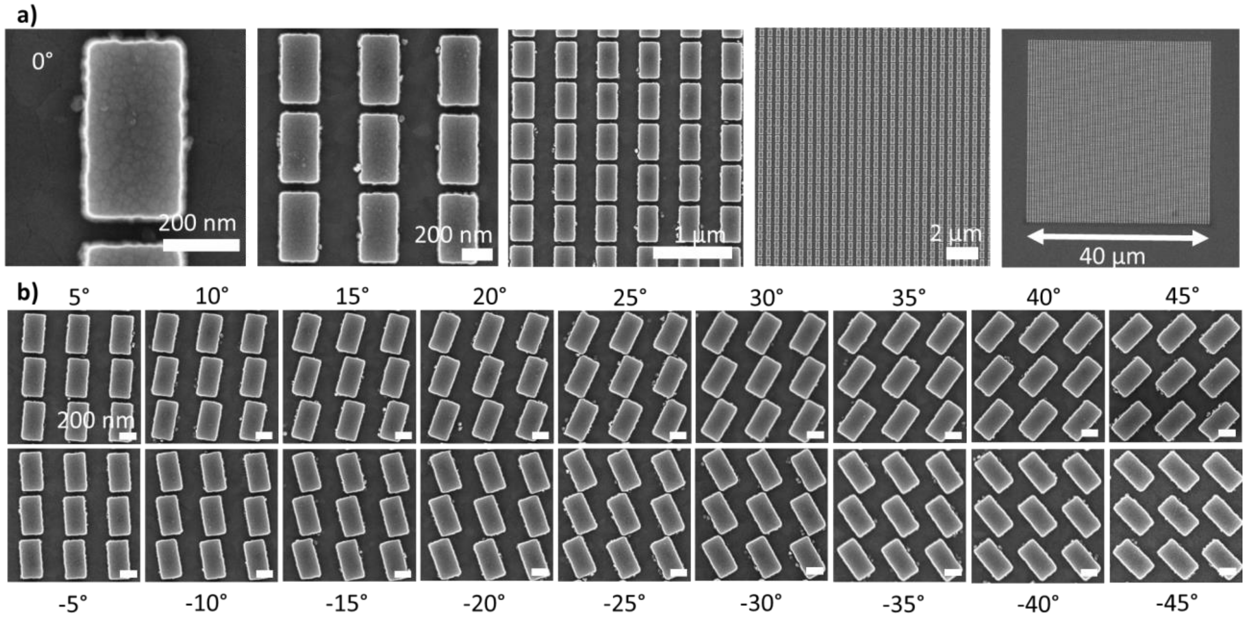

Figure S2: Scanning Electron Microscopy (SEM) images of the fabricated metasurfaces.
(a) SEM images of the achiral metasurface with a rotation angle of 0° at different magnifications. The total size of all metasurfaces was 40 µm x 40 µm with 70 x 70 unit cells. **(b)** SEM images of all fabricated metasurfaces with different rotation angles from -45 ° to +45 ° in 5° steps. The scale bar is 200 nm.

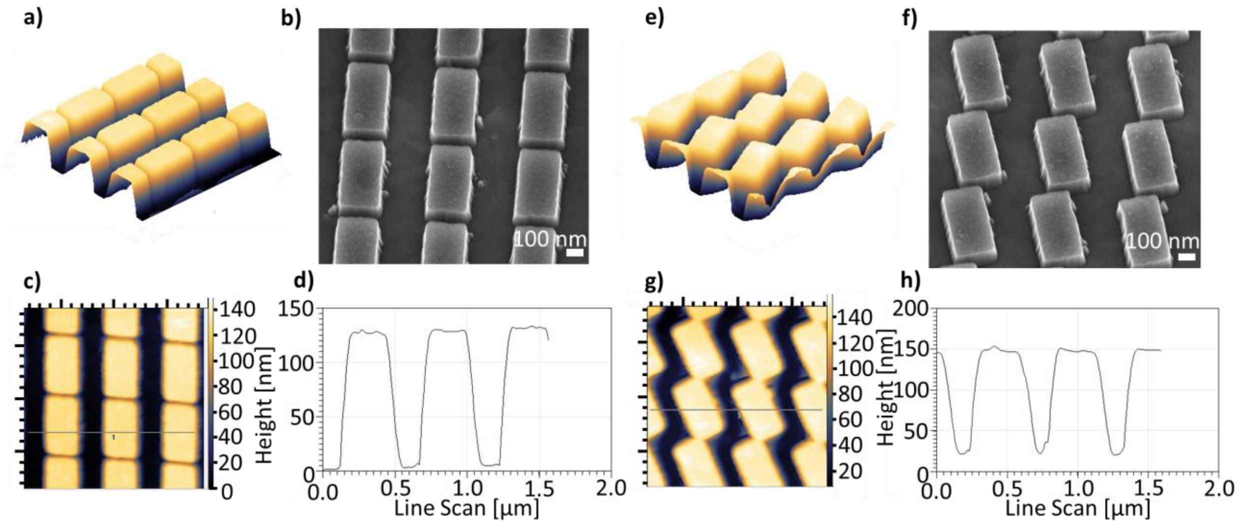

Figure S3: Atomic Force Microscopy (AFM) images.

(a-d) Achiral metasurface with rotation angle of 0°. **(e-h)** Chiral Metasurface with rotation angle of 25°. (a,e) 3D AFM micrographs. (b, f) Tilted SEM images (tilt angle 30°). (c, g) AFM top view. (d,h) Line Scan along the profile shown in (c,g).

Section 3 - Details on the optical setup

Optical characterization was performed following other studies on chiral metasurfaces reported previously (see also the setup figures in the Supporting Information).[4]

Briefly, a custom-built transmission microscope was used on an optical table with a supercontinuum white light laser (SuperK Extreme from NKT Photonics) as the illumination source with a power of 5 % of the maximum value and a repition rate of 0.302 MHz. Horizontal (HP) or vertical (VP) linear polarization was generated using a broadband linear polarizer (LPVIS100 from ThorLabs, 550 – 1500 nm) and circularly polarized light (CPL) was generated using a broadband quarter-wave plate (QWP, RAC4.4.20 from B-Halle, 500-900 nm). The QWP was located directly under the microscope objective (Olympus PLN, 10×, NA = 0.25, which served as a condenser) to avoid reflections from mirrors, which can change the CPL purity degree of the illumination.

Light was condensed on the sample by the microscope objective and collected with a 60× objective (Nikon MRH08630, NA = 0.7). After alignment, the beam was slightly defocused to illuminate the entire metasurface (SEM images in Figure S2), and an aperture was used to select the light coming from the area occupied by the metasurface, minimizing the detection of any background illumination, that is, light which hasn't interacted with the metasurface. The light was then sent to a CCD camera or to a multimode fiber (ThorLabs M15L05, core size: 105 µm, NA = 0.22) connected to a grating spectrometer (Princeton Instruments, 300 g/mm grating period, blazed at 750 nm, 0.13 nm spectral resolution).

Cross- and co- polarized transmission measurements were performed by using a chiral analyzer consisting of a QWP (AQWP05-580 from Thorlabs, 350 – 850 nm) and a linear polarizer

(WP25M-UB from Thorlabs, 250 – 4000 nm). The chiral analyzer was installed directly after the collection objective.

Section 4 - Details on the optical characterization

All spectra were acquired with a binning of one CCD line, an exposure duration of 40 ms and 40 spectra were accumulated. All spectra were referenced using a background measurement made on the same substrate far from the metasurface using the same illumination and spectral detection conditions.

Our measurements on the achiral metasurface and measurements for other projects[4] revealed a small degree of elliptical polarization in our setup. The elliptical polarization is well explained by the manufacturer's stated deviation of the QWP and is stronger at wavelengths > 900 nm, which is somewhat outside its operating range. This elliptical polarization manifests itself in a chiral signal for the achiral metasurface that reverses its sign when the substrate is rotated by 90° (**Figure S4**). To account for this, all measurements shown in the manuscript were repeated twice with a 90 ° rotation of the substrate and their average was taken.

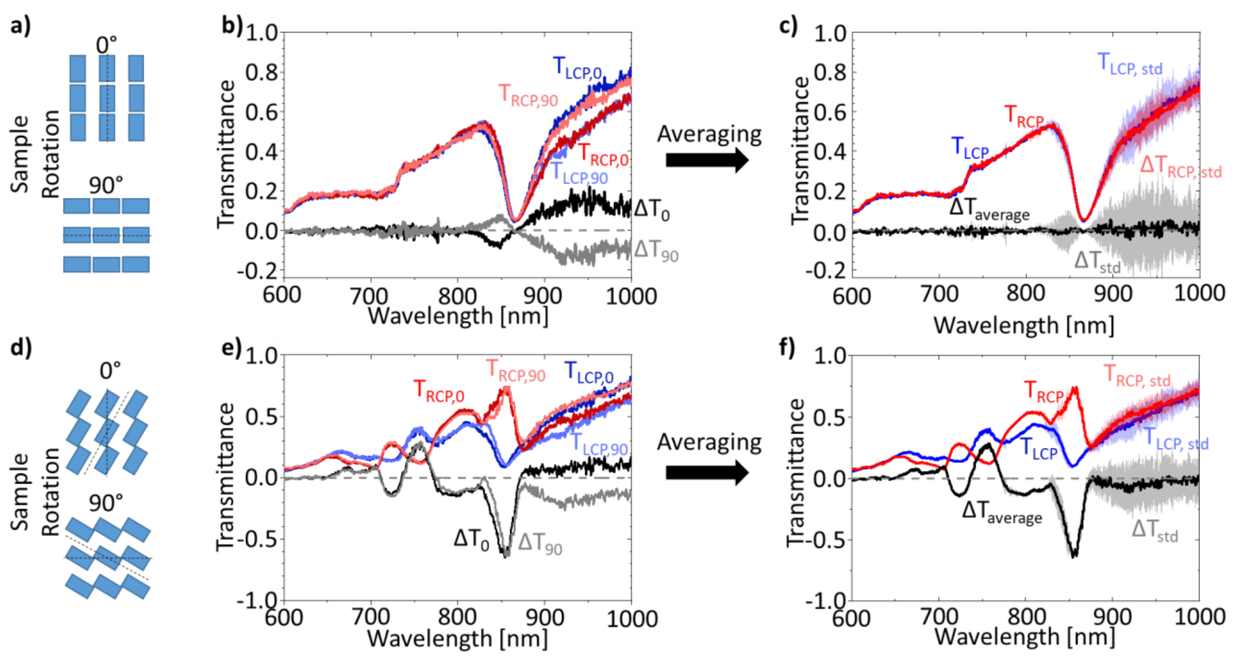

Figure S4: Processing of the optical characterization.
Processing of the optical characterization by averging. **(a-c)** Achiral metasurface with rotation angle 0°. **(d-f)** Chiral metasurface with rotation angle 25°. All spectra were recorded twice with a

90° rotation of the substrate (Schematic illustration on the left side). (b,e) Each set of measurements shows a distinct chiral signal, especially >900 nm, that reverses sign when the substrate is rotated by 90°, indicating elliptical polarization. (c,f) Average signal and standard deviation (std, shown as shaded area) from both measurements. All experimental spectra shown in the manuscript were recorded using this method.

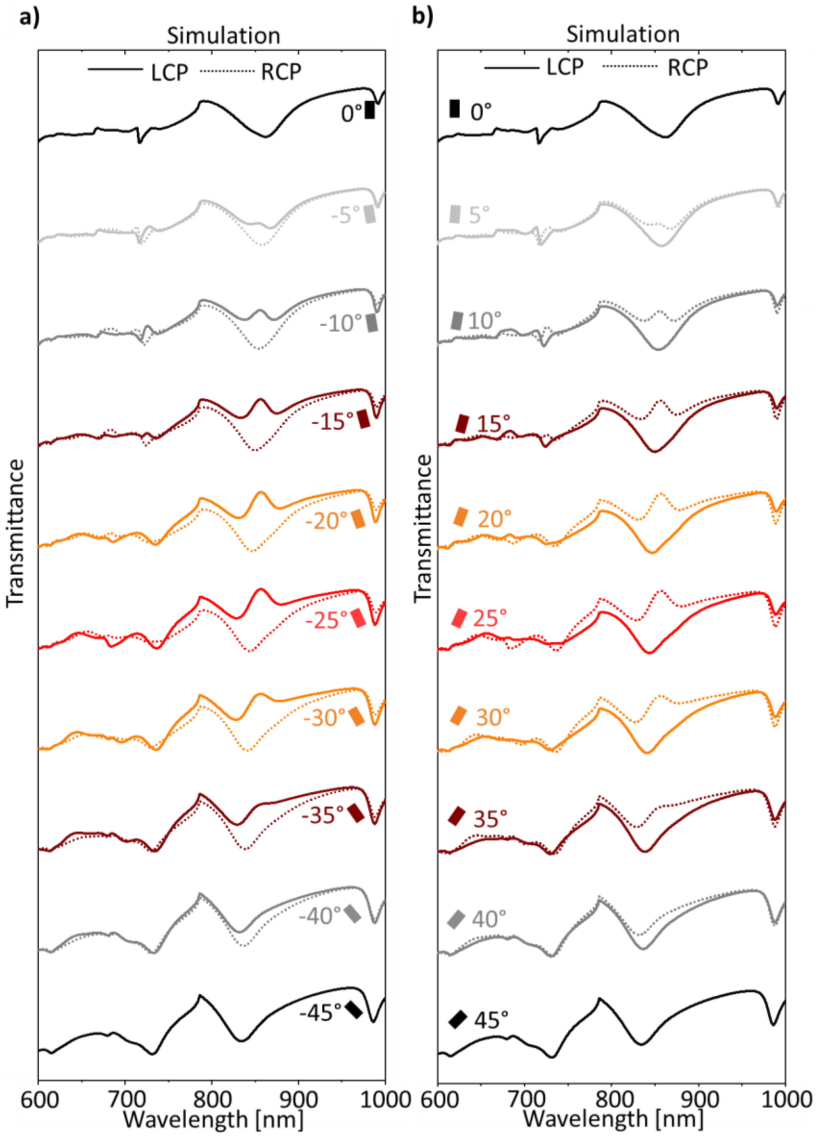

Figure S5: Simulated transmission spectra for LCP and RCP illumination for all rotation angles.

Simulated transmission spectra for rotation angles from 0 to -45° **(a)** and 0 to +45° **(b)**. LCP illumination is shown as solid line, RCP as dotted line. The spectra have been offset for clarity. The light interaction is perfectly mirrored for the same clockwise and counterclockwise rotation. Experimental transmission spectra are shown in Figure S6.

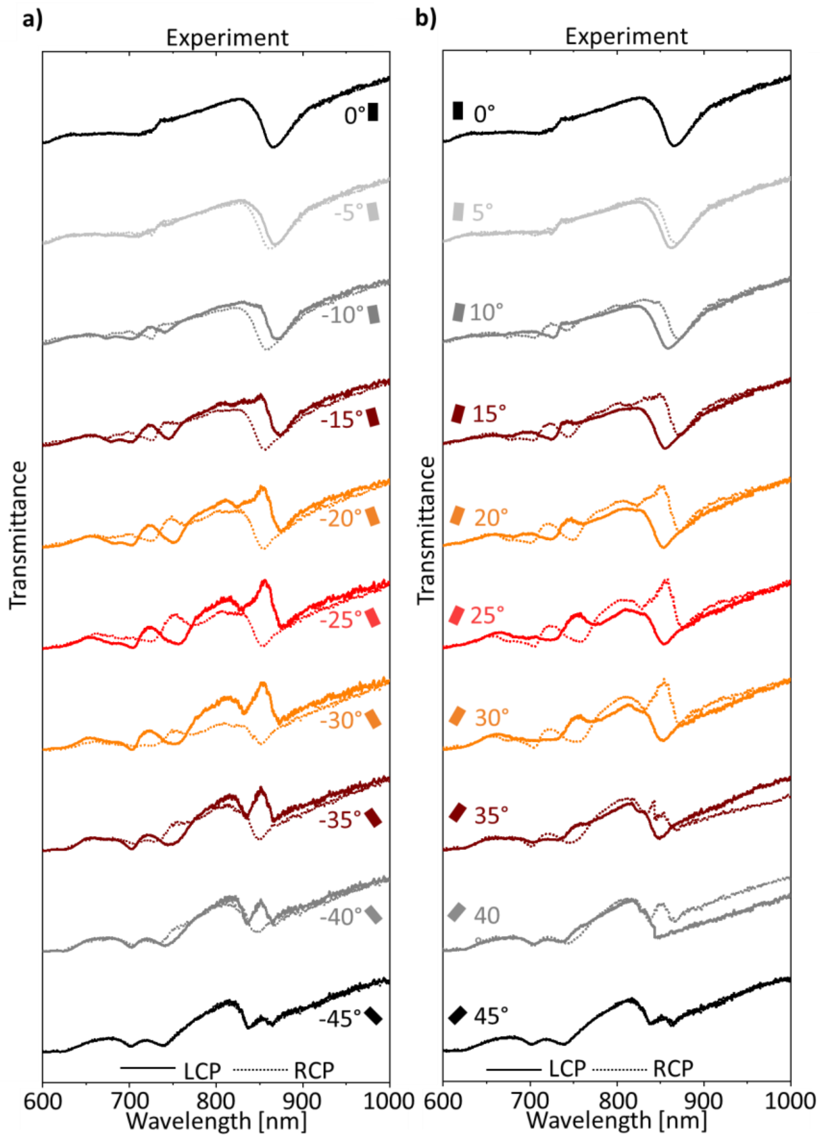

Figure S6: Experimental transmission spectra for LCP and RCP illumination for all rotation angles.

Experimental transmission spectra for rotation angles from 0 to -45° **(a)** and 0 to +45° **(b)**. LCP illumination is shown as solid line, RCP as dotted line. The spectra have been offset for clarity. The light interaction is perfectly mirrored for the same clockwise and counterclockwise rotation. Simulated transmission spectra are shown in Figure S5.

Section 5 – Excitation with linear polarization.

In an attempt to explain the origin of the observed chiral resonances, we simulated the transmission spectra of all metasurfaces illuminated with linearly polarized light. Circularly polarized light can be interpreted as two linear polarized waves with polarizations perpendicular to each other and having a phase difference of π/2 (LCP) or -π/2 (RCP). Thus, looking at linear polarizations allows us to decouple the interaction of the structure with CPL, which makes it easier to relate the resonances to the structure. For example for the achiral case with rotation angle of 0° some resonances only occur for one linear polarization, but not for the other. However, the influence of the phase difference between RCP and LCP light cannot be taken into account with this approach. For all simulations, we used horizontal and vertical linear polarization, HP and VP, respectively, which remain in the same orientation during meta-atom rotation. First, we performed small parameter sweeps for the geometric dimensions of the achiral metasurface with rotation angle of 0° (**Figure S7**). Then, we simulated all metasurfaces with different rotation angles (**Figure S8**). All resonances are labeled with numbers and symbols to facilitate the discussion.

<u>Parameter sweeps:</u>

We simulated the achiral sample with rotation angle of 0° and exclusively varied one parameter, i.e. length, width or pitch, while keeping the other parameters constant. The results are shown in Figure S7. Beginning with HP, we can see that resonances 1#, 2#, 3# 6# are exclusively shifting for variation of the structures, i.e. length and width, while they remain stable for pitch variation. Resonance 4# is exclusively dependent on the pitch, while resonance 5# varies with both width and pitch. For VP, we can see that 7#, 8# are exclusively shifting for variation of the structure, i.e. length and width, while resonance 9#, 10# depend on all parameters and resonance 11# is exclusively depending on the pitch. *Note: Resonance 10# varies in modulation strength for length*

(a) and width (b) variation, but does not experience any obvious shift in wavelength. Similarly Resonance 8# varies for pitch variation, but only experiences a small shift in comparison to resonance 10# and 11#.

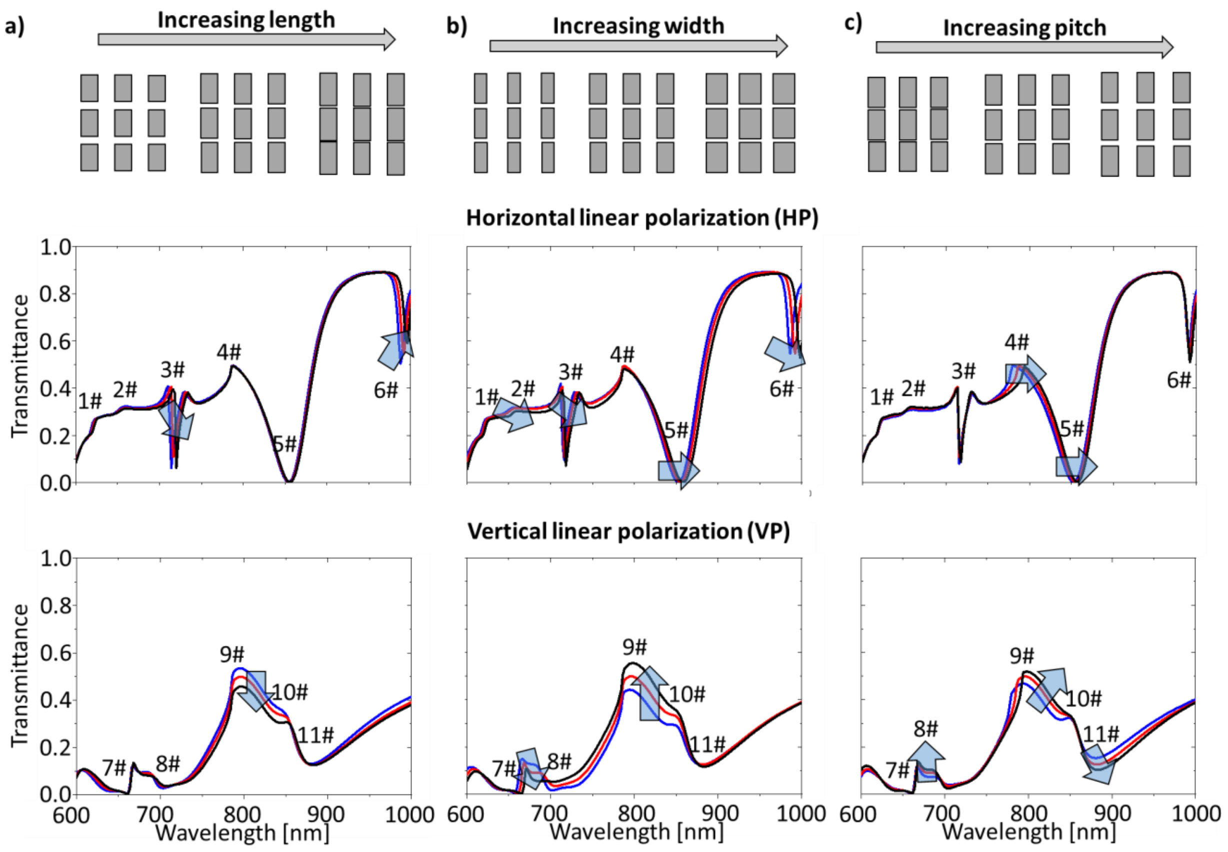

Figure S7: Parameter variation of the structures dimensions with horizontal and vertical linear polarization.

Changes in the transmission spectra for the achiral sample with rotation angle of 0°. Top: Schematic illustration. Middle: illumination with horizontal linear polarization (HP). Bottom: illumination with vertical linear polarization (VP). **(a)** Increasing the rods length from 468 to 478 nm in 5 nm steps. **(b)** Increasing the rods width from 242 to 252 nm in 5 nm steps. **(c)** Increasing the pitch from 535 to 545 nm in 5 nm steps. The results are discussed in SI Section 5.

Different rotation angles:

Next, we simulated all metasurfaces with different rotation angles with HP and VP excitation (Figure S8). We can see that several resonances are shifting during the rotation. These shifts might be addressed to changes in the excited dimensions as illustrated in schemes in Figure S8 on the right. For example, HP experiences a "widening" of the structure (~width) upon further rotations (indicated as dark blue), whereas the distance between the structure (~gap) decreases (indicated as light blue). For VP such an interpretation is more difficult, since the excited dimensions experience a change upon rotation, since their "length" first becomes longer and then after the rod is rotated over the corner, becomes shorter. Similarly, the "gap" first becomes shorter and then longer. For +/- 45° HP and VP are equal. However, due to the large number of features, this interpretation is quite complex and requires a more in-depth analysis.

Resonance 4# (9#) can be clearly related to the pitch, since it does not shift at all (there is no change in pitch during rotation) and is present for both HP and VP. For HP, we can see that resonances 2#, 3# are blueshifting so they might be related to the structures dimensions. Resonance 5# blueshifts and might therefore be connected to the gap size. For VP, resonance 7# and 11# might be connected to the structure, since they first redshift until 25° and then blueshift.

Interestingly, even though many resonances experience wavelength shifts with HP and VP polarization under the sample rotation, their ΔT signal is not shifting at all (Figure 2 in the main text). This means, that under LCP and RCP excitation, all these wavelength shifts perfectly compensate each other and the ΔT signal originates mainly from the phase difference induced by the meta-atom rotation.

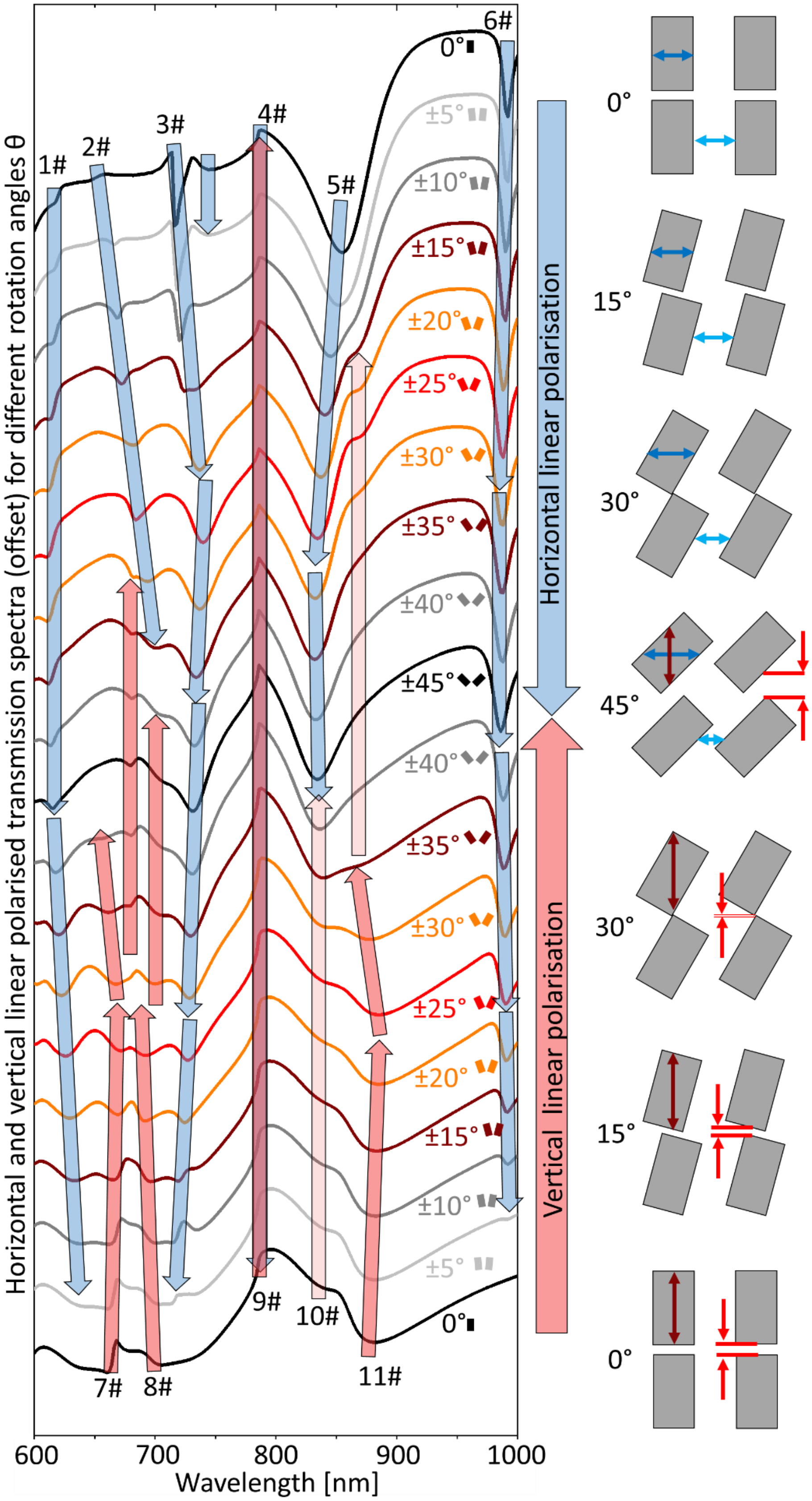

Figure S8: Simulated transmission spectra for horizontal and vertical linear polarisation. Horizontal and vertical linear polarisations, HP and VP, respectively are equal for positive and negative rotation angles. HP is shown at the top half of the figure and rotation angles are increasing downwards. VP is shown at the bottom half of the figure and rotation angles are increasing upwards. For ±45°, HP and VP are equal.

Left: Simulated transmission spectra, the blue (red) arrows indicate the progression of peaks originating from the HP (VP) during the meta-atom rotation. Lighter blue (red) arrows indicate resonances, which are only barely visible.

Right: Schemes illustrating the changes upon meta-atom rotation in respect to HP (in blue) and VP (in red). As example, for HP the width of the structure is increasing for HP (dark blue arrows), while the gap size decreases (light blue arrows) for rotations from 0 to 45°

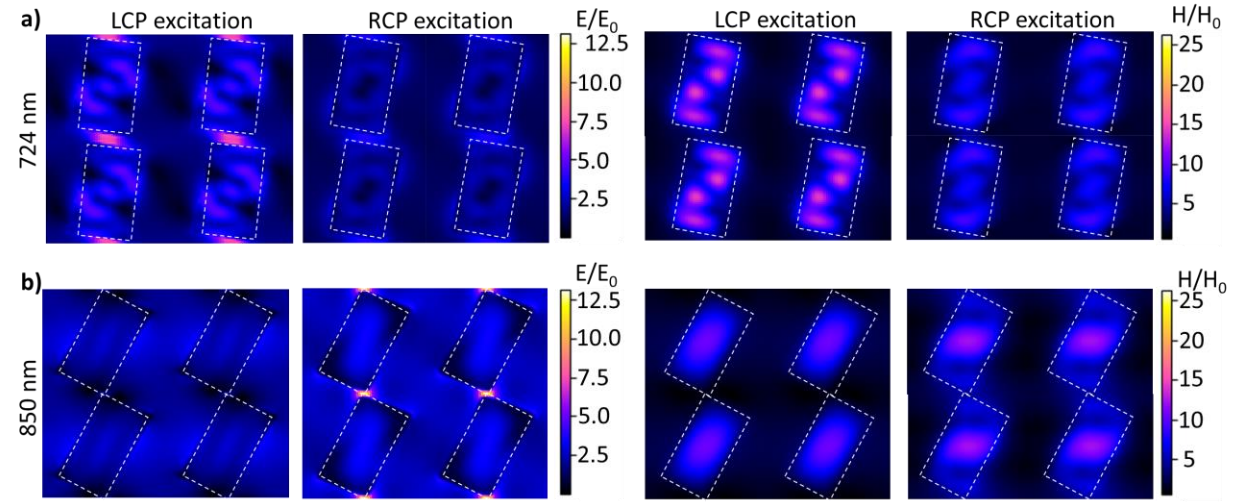

Figure S9: Electric and magnetic near-field plots for the metasurfaces with rotation angle of 10° and 25° at their resonance wavelength.

Electric (left) and magnetic (right) near field plots at resonance wavelength with rotation angles of 10° (a) and 25° (b) for LCP and RCP excitation. The electric and magnetic fields have been normalized the electric and magnetic field of the illumination source, E_0 and H_0, respectively.

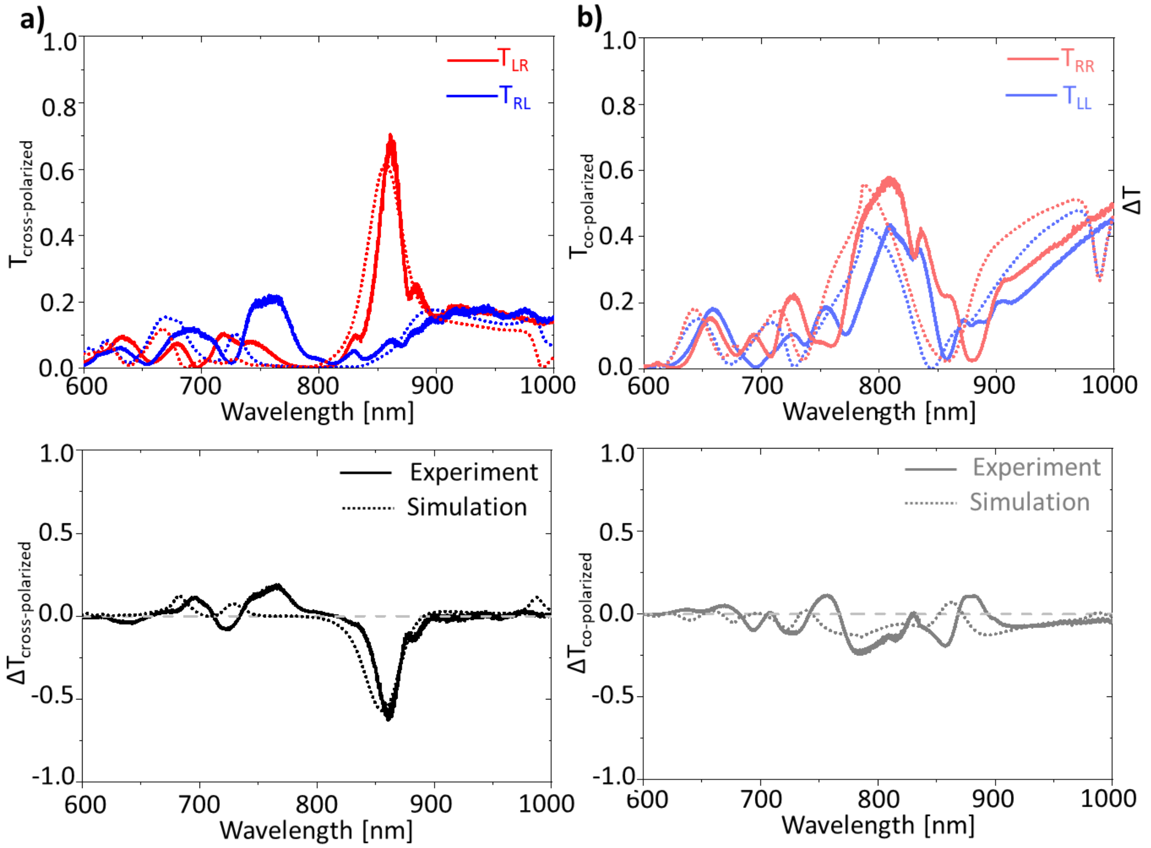

Figure S10: Cross- and co-polarized transmission and transmission asymmetry.
Cross- and co-polarized transmission was measured by employing a chiral analyzer to characterize the output polarization of the transmitted light, which allows to distinguish between the effects of 2D- and 3D-chirality, respectively. (a,b) Top: Experimental (solid line) and simulated (dotted line) cross- and co-polarized transmission. Bottom: Experimental (solid line) and simulated (dotted line) cross- and co-poralized transmission asymmetry. The main peak at 850 nm is mostly dominated by 2D chirality.

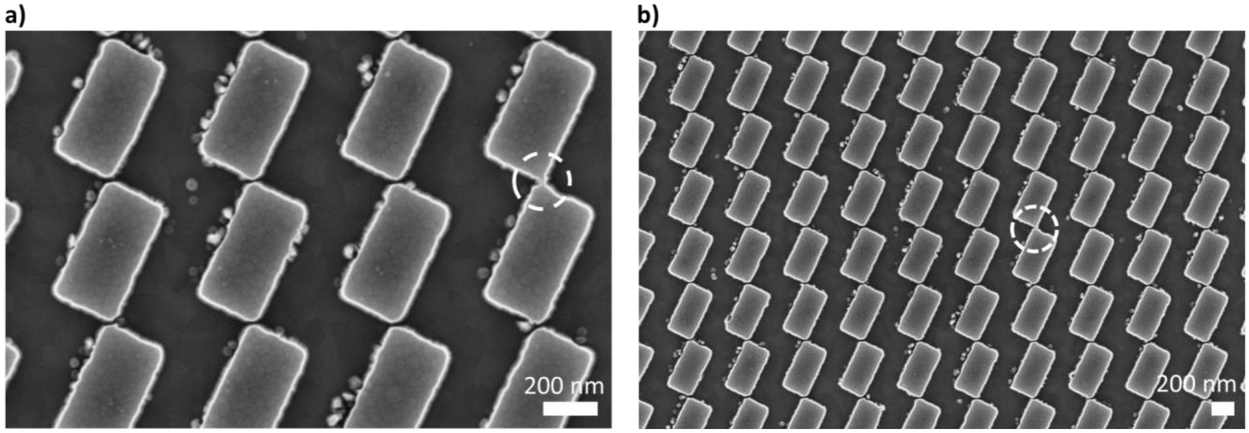

Figure S11: Gap sizes in the experimental realization.
(a,b) SEM images at different magnifications showing the realized gap sizes. The measurement of 60 gaps resulted in an average gap size of (13.6 ± 3.1) nm. Only one out of the 60 gaps was bridged, which is visible at the top right (dashed white circle).

Section 6 – Geometric asymmetry.

To characterize the geometric asymmetry, we calculated the geometric overlap of our meta-atoms with their mirror images for different rotation angles and analyzed at which rotation angles the geometric overlap of the structures is minimal, i.e., maximum asymmetry. A graphic representation can be found in Figure S12. We began with calculating the "normal" overlap of the structures (blue line in and schematic illustrations at the top of Figure S12). The structures are perfectly overlapping at 0° rotation angle, leading to a normalized geometric overlap 1.0. For positive and negative meta-atom rotations the overlap decreases and gets minimal for ±45°. Since chiral objects cannot be brought into coincidence by any lateral and translational operations, we performed the same calculation with a 90° rotation of one of the meta-atoms (red line in and schematic illustration at the bottom of Figures S12). At 90° rotation of one of the meta-atoms, the previous minimum geometric overlap at ±45° results in a perfect overlap, while the previous maximum at 0° becomes the minimum geometric overlap. The geometric asymmetry lies in between these two extremes and is illustrated as gray shadow in Figure S12, featuring a symmetric shape with the maximal geometric asymmetry at 22.5°.

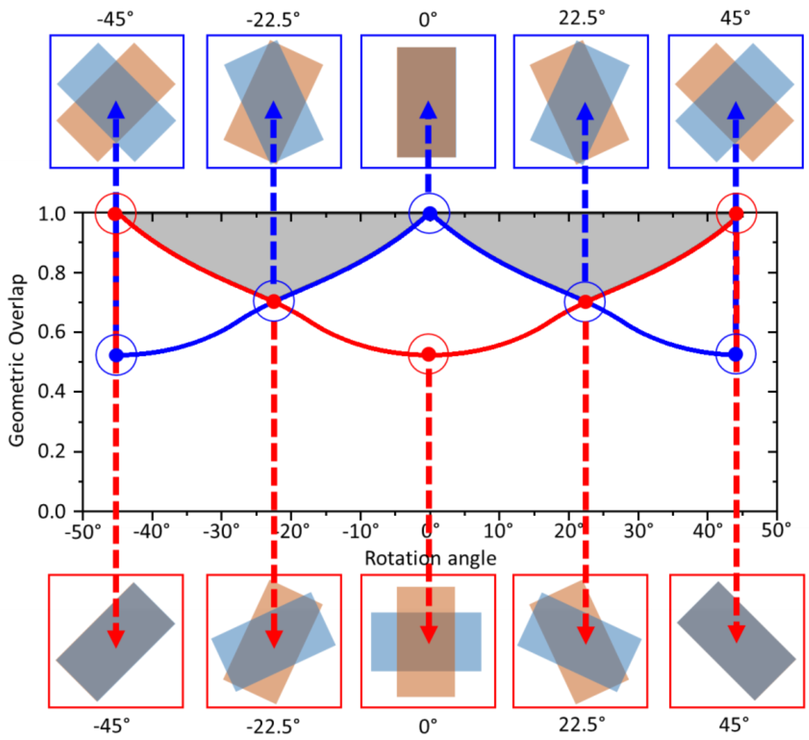

Figure S12: Asymmetry calculation of the metasurfaces with different rotation angles.
Top: Graphic illustration of the geometric overlap of the metasurface with different rotation angles. The overlap is maximal for 0° and minimal for ± 45°, shown as blue line in the graph. Bottom: Graphic illustration of the geometric overlap with a 90° rotation of the meta-atoms. The overlap is maximal for ± 45° and minimal for 0°, shown as red line in the graph. For both, red and blue lines, the maximum overlap has been normalised to 1.0. The asymmetry is shown as black shaded area and is maximal for a rotation angle of 22.5°.

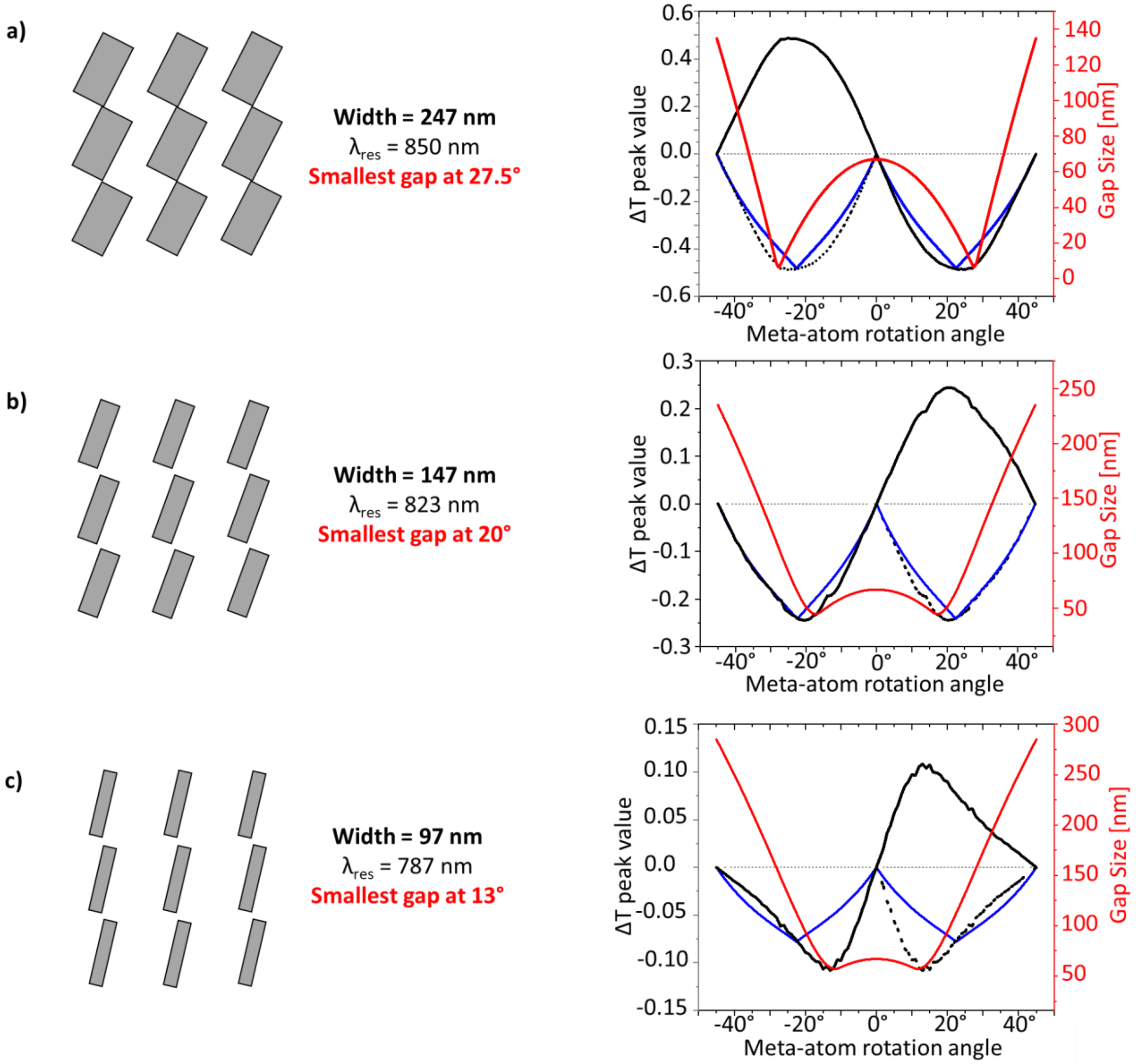

Figure S13: Comparison of the chiroptical response with the geometric assymetry and gap size for different meta-atom rotation angles and widths.
a-c) Structures with decreasing width to decouple the geometric asymmetry from the gap size. Left: Schematic illustration. Right: Comparison of the peak transmission asymmetry ΔT (black line) with the geometric asymmetry (blue line) and the gap size (red line) for different meta-atom rotation angles. The positive chiroptical response has been mirrored (dashed black line) for clarity.

The values of the gap sizes are shown on the right y-axis, while the values of the geometric assymmetry are arbitrary. Their shape was taken from Figure S12 and was scaled that their minima (which corresponds to maximum geometric asymmetry) hits the spectra of transmission asymmetry in the graph to allow a better comparison. The values of the gap size have also been

adjusted, i.e. the right y-axes do not start at zero, so that the minimum gap size also hits the spectra of transmission asymmetry for the same reason.

The geometric asymmetry (in blue) is unaffected by changes in width, shows its maximum asymmetry at 22.5° (for rods) and mainly determines the global chiroptical response, i.e. $\Delta T = 0$ at ± 45° and 0°. The meta-atom rotation angle with maximum transmission asymmetry shifts to smaller angles with decreasing width (black line), which is in correlation to the minimum gap size (in red), which shows the same behaviour.

References

(1) Palik, E. D.; Ghosh, G. *Handbook of Optical Constants of Solids*; Academic Press: San Diego, 1998.

(2) Moerland, R. J.; Hoogenboom, J. P. Subnanometer-Accuracy Optical Distance Ruler Based on Fluorescence Quenching by Transparent Conductors. *Optica* **2016**, *3* (2), 112. https://doi.org/10.1364/OPTICA.3.000112.

(3) Vinçon, I.; Wendisch, F. J.; De Gregorio, D.; Pritzl, S. D.; Akkerman, Q. A.; Ren, H.; de S. Menezes, L.; Maier, S. A.; Feldmann, J. Strong Polarization Dependent Nonlinear Excitation of a Perovskite Nanocrystal Monolayer on a Chiral Dielectric Nanoantenna Array. *ACS Photonics* **2022**, *9* (11), 3506–3514. https://doi.org/10.1021/acsphotonics.2c00159.

(4) Kühner, L.; Wendisch, F. J.; Antonov, A. A.; Bürger, J.; Hüttenhofer, L.; Menezes, L. de S.; Maier, S. A.; Gorkunov, M. V.; Kivshar, Y.; Tittl, A. Unlocking the Out-Of-Plane Dimension for Photonic Bound States in the Continuum to Achieve Maximum Optical Chirality. **2022**. https://doi.org/10.48550/ARXIV.2210.05339.
